# Fluorescence Based Comparative Sensing Behavior of the Nano-Composites of SiO_2_ and TiO_2_ towards Toxic Hg^2+^ Ions

**DOI:** 10.3390/nano11113082

**Published:** 2021-11-15

**Authors:** Ekta, Divya Utreja

**Affiliations:** Department of Chemistry, Punjab Agricultural University, Ludhiana 141004, India; iektamalhotra@gmail.com

**Keywords:** solid-state lighting, optical sensing, optical nanomaterials, Hg^2+^ ions

## Abstract

We have synthesized sulfonamide based nano-composites of SiO_2_ and TiO_2_ for selective and sensitive determination of toxic metal ion Hg^2+^ in aqueous medium. Nano-composites **(11)** and **(12)** were morphologically characterized with FT-IR, solid state NMR, UV-vis, FE SEM, TEM, EDX, BET, pXRD and elemental analysis. The comparative sensing behavior, pH effect and sensor concentrations were carried out with fluorescence signaling on spectrofluorometer and nano-composites **(11)** and **(12)**, both were evaluated as “turn-on” fluorescence detector for the toxic Hg^2+^ ions. The LODs were calculated to be 41.2 and 18.8 nM, respectively of nano-composites **(11)** and **(12)**. The detection limit of TiO_2_ based nano-composites was found comparatively lower than the SiO_2_ based nano-composites.

## 1. Introduction

Among the several metal ion-based pollutants, mercury is a frontier contaminant to the human health and environment. Both natural and anthropogenic activities generate mercury contaminations in the surroundings [1,2,3,4]. Mercury in all of its oxidation states (Hg_2_^2+^, Hg^2+^, Hg^0^, [CH_3_Hg]^+^) is released in the environment by combustion of coal, medical and industrial waste [5,6,7]. Additionally, as a consequence of processes such as chlor-alkali and gold mining add mercury into the nature. In addition, the inorganic mercury pollutants possess capability to absorb and transform into organic ones by bacteria and microbes [8,9,10,11,12]. The most abundant and stable forms of mercury present in nature, are in its +2 oxidation state. Mercury enters in living system through respiration, skin absorption and oral take-up. Owing to its high bioaccumulation and bio-amplification, multi-step contamination of food chain to hazardous level of mercury has been reported [13,14,15]. Its small amount in the body triggers the long-term irreversible damages to the human health by incorporating the unfavorable impact on the vital organs and tissues such as brain, nervous/immune system, kidney, liver and induces the cognitive and motion disorders [16,17,18,19,20]. The maximum permissible concentration of Hg^2+^ ions is 1 µg/L in drinking water, which is defined by the United States Environment Protection Agency [21]. Therefore, it is of great importance to develop a rapid and eco-friendly method to detect Hg^2+^ ion with high sensitivity and selectivity. 

Atomic-absorption spectrometry (AAS) and inductively-coupled plasma mass spectrometry (ICP-MS) are the most common instrumental techniques for metal detection but colorimetric and fluorogenic sensing procedures are found to be more effective and on-site units for this purpose [22,23]. The colorimetric method is advantageous because of easy readout with the naked eye and potential for high throughput formats. Being organic in nature, these sensors are sometimes associated with certain limitation such as less stability, use of high concentration and also do not have a limit of detection (LOD) as low as a fluorescent- or luminescent-based approach [24,25,26,27,28]. Moreover, these are incapable of removing the ions from the medium owing to their diffusive nature which is further rectified with nano-tool technique. 

Nano-tools (nanoparticles/nano-composites) of different compositions and morphologies have attracted immense attention due to their low LOD, intact composition and solid-phase-sensing ability [29]. The optical properties of nanoparticles differ from those of bulk materials because nano-materials have size dependent effects that make their absorption and scattering unique [30]. Therefore, combination of nano-materials with well established chemistry results in the formation of nano-tools for biochemical and chemical assays. Recently functionalized nano-particles witnessed several applications in drug delivery, bio-imaging and catalysis etc [31,32,33,34]. These are proved as important types of binding hosts for developing functional materials due to their large surface area, high thermal stability and uniform porosity. The incorporation of the organic ligand on the solid surfaces such as silica (SiO_2_) and titania (TiO_2_) proved as a prevailing tool for building inorganic and organic nano-composites with improved optical properties [35,36]. The scattering properties of nano-materials depend on the composition, size and shape of nanoparticles and their surrounding medium. The scattered light intensity by nanoparticles has been proved to increase by the sixth power of the particle size and hence, modifying and enhancing the size of nanoparticles help to increase the intensity of scattered light and immobilization with the organic moieties further help in acquiring a target analyte [37,38,39,40].

Hence, in the present work nano-particles of SiO_2_ and TiO_2_ were taken as building blocks for adhering the synthesized organic ligand **(3)**. Since these are non-fluorescent and provide large surface area due to abundant Si-(OH)_n_ and Ti-(OH)_n_ sites on the nano-particles’ surface for the attachment of organic ligand via electrostatic interactions between fluorophore **(3)** and the amino group of functionalized SiO_2_ and TiO_2_. The prepared nano-composites were found to detect only toxic Hg^2+^ ions, out of all other tested ions. The emission spectral data was recorded to analyze the sensing ability of nano-composites **(11)** and **(12)** for Hg^2+^ detection in aqueous samples and their LODs were found to be 41.2 and 18.8 nM, respectively. It is noteworthy that even on the coating of same organic ligand **(3)** on the functionalized SiO_2_ and TiO_2_ surfaces there is a visible difference between the detection limits of the Hg^2+^ ions. Titania nano-composites **(12)** were found to be more effective for detection of Hg^2+^ ions as compared to silica nano-composites **(11)** in aqueous medium.

## 2. Materials and Methods

Commercially available analytical grade reagents and solvents without further purifications were used. 3-Formyl chromone and sulfanilamide were procured from Sigma Aldrich (St. Louis, MO, USA) with >99% purity. Tetraethoxysilane (TEOS), tetraisopropylorthotitanate (TIPT) and 3-aminopropyl triethoxysilane were purchased from Tokyo Chemical Industry (TCI) Co. Ltd., Tokyo, Japan and were used without further purification. Salts of metal nitrates and sodium salts of anions were purchased from Sigma Aldrich and Hi-Media Laboratories Pvt. Ltd. (Mumbai, India). Pyrogen free distilled water was obtained from distilled water assembly made from borosilicate glass by Jain Scientific Glass works (Ludhiana, India). Melting point was determined using Nutronics digital melting point apparatus available at Central Instrumentation Laboratory, Punjab Agricultural University (PAU), Ludhiana, India in open capillary and is uncorrected.

Fluorescence spectra were recorded using Agilent Cary Eclipse Fluorescence spectrophotometer with excitation and emission wavelength band passes of 2.5 nm from FIST Laboratory, Department of Biochemistry, PAU, Ludhiana, India. SHIMADZU 1800 PC spectrophotometer (Akishima, Japan) in the range 200–1100 nm with quartz cuvettes (path length, 1 cm) was used to perform all UV-vis spectral analysis at Central Instrumentation Laboratory, PAU, Ludhiana, India. FT-IR spectrum of the ligand and nano-composites were recorded on a Perkin Elmer Spectrum Two-IR Fourier-Transform spectrophotometer (Waltham, MA, USA) in the range 400–4000 cm^−1^ (KBr pellets). The ^1^H NMR and ^13^C NMR spectra of the ligand **(3)** were recorded in DMSO on a BRUKER 500 MHz and 125 MHz spectrometer respectively (Fällanden, Switzerland), at room temperature using TMS as an internal standard and chemical shifts are given in δ. Solid State ^13^C and ^29^Si cross-polarization magnetic angle spinning (CPMAS) NMR were recorded at Bruker 700 MHz spectrometer at Tata institute of Fundamental research (TIFR) Centre for interdisciplinary Science, Hyderabad, India. The surface morphologies of the samples were determined by Field Emission Scanning Electron Microscope (FESEM, Hitachi, Ibaraki Prefecture, Japan). Samples were recorded on Hitachi SU 8010 with EDX (Thermo Noran System SIX, Ibaraki Prefecture, Japan). The X-ray diffraction spectroscopy (XRD) patterns were recorded in the range of 5°–85° 2θ, on a SAXSPACE, Anton Paar instrument (Gurugram, India), provided with a Cu-K_α_ radiation source (*λ* = 0, 154,060 nm). FT-IR, NMR, FE SEM and Powder XRD studies conducted at Sophisticated Analytical Instrumentation Facility (SAIF), Panjab University (PU), Chandigarh, India.

Brunauer–Emmett–Teller (BET) surface area was determined using a Quanta Chrome Autosobe iQ3 instrument (Cinnaminson, NJ, USA) from Advanced Material Research Centre situated at Indian Institute of Technology (IIT), North Campus, Mandi, Himachal Pradesh. Surface area and pore volume were determined using the BET equation and Barret-Joyner-Halenda (BJH) methods, respectively. 

Stock solutions (10^−4^ M) of cations Al^3+^, Ag^+^, Ba^2+^, Ca^2+^, Cd^2+^, Cu^2+^, Cr^3+^, Co^2+^, Fe^2+^, Fe^3+^ Hg^2+^, Hg^+^, K^+^, Li^+^, Mn^2+^, Mg^2+^, Na^+^, Ni^2+^, Pb^2+^ and Zn^2+^ (nitrate salts) and anions AcO^−^, Br^−^, BO_3_^−^_,_ CO_3_^2−^, C_2_O_4_^2−^, Cl^−^, F^−^, HSO_3_^−^, HPO_4_^2−^, HCO_3_^−^, I^−^, NO_3_^−^, NO_2_^−^, OH^−^, SO_4_^2−^ and SO_3_^2−^ (sodium salts) were prepared in double distilled water. Stock solutions of the nano-composites **(11)** and **(12)** were also prepared by dispersing 0.01 g nano-composites in 1.00 L of double distilled water. Further, these dispersed solutions of various synthesized nano-composites were sonicated for an hour to obtain a stable colloidal solution. 3.00 mL of appropriate aliquot were taken in a quartz cuvette, in which 50.00 nM solution of various metal ions and anions were added sequentially to check the selectivity of nano-composites for any specific ion.

Finally, the practical utility of the nano-composites **(11)** and **(12)** for Hg^2+^ ion detection on tap water sample, distilled water and bottled water was checked. The tap water was taken from research laboratory of Department of Chemistry (PAU, Ludhiana) and bottled water was purchased from local market. These collected samples were filtered and adjusted to pH 7.4 (10 mg in 5 mL HEPES buffer). These samples were spiked with various concentrations of Hg^2+^ ions. The fluorescence intensities were recorded in triplets with their mean values as final datum to calculate the percentage recovery.

### 2.1. Synthesis of 4-((4-Oxo-4H-chromen-3-yl)methyleneamino)benzenesulfonamide ***(3)***

**Scheme 1:**
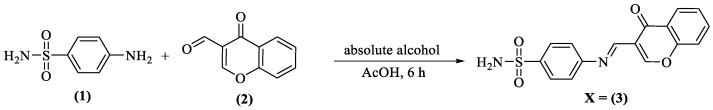
Synthesis of 4-((4-oxo-4*H*-chromen-3-yl)methyleneamino)benzene sulfonamide **(3)**.

For the preparation of the Schiff’s base ligand **(3)**, sulfanilamide **(1)** (1.00 mmol, 0.174 g) and 3-formyl chromone **(2)** (1.00 mmol, 0.172 g) were taken in a round bottomed flask containing 15.00 mL of absolute alcohol (Scheme 1). After 5 min, 2–3 drops of glacial acetic acid (AcOH) were added and the mixture was refluxed until the completion of reaction (6 h, TLC). Further, reaction mixture was allowed to stand at room temperature and solid so obtained was filtered and washed with diethyl ether (3 × 40.00 mL). Recrystallization from absolute alcohol furnished the pure product **(3)**.

Analytical data: Shiny yellow colored solid, yield: 91%, melting point: 302–303 °C, Solubility: absolute alcohol, ^1^H NMR (DMSO-*d_6_*, 500 MHz) *δ* (ppm): 5.84 (s, 1H, OC-H), 7.11–7.17 (m, 2H, Ar-H), 7.29 (s, 2H, SO_2_NH_2_), 7.53–7.85 (m, 5H, Ar-H), 8.16 (s, 1H, Ar-H) and 8.18 (s, 1H, -CH=N) ppm. ^13^CNMR (DMSO-*d_6_*, 125 MHz) δ: 101.05, 104.56, 116.17, 116.35, 118.05, 122.00, 122.40, 125.59, 127.34, 127.50, 134.71, 138.76, 142.42, 144.23, 155.46 and 180.51 ppm. IR (KBr) υ_max_: 1069 (C-O str), 1160 (C-S str), 1212 (C-N str), 1390 (S=O str), 1472 (C=C str), 1596 (C=O str), 1656 (C=N str), 3077 (=C-H str) and 3308.0 (N-H str) cm^−1^. MS (ESI-MS): calculated for C_16_H_11_N_2_O_4_S *m*/*z* (M^+^+1) 328.05; found, 329.07. (Figure S1a–d)

### 2.2. Synthesis of Nano-Composites of SiO_2_ ***(7)*** and TiO_2_ ***(9)***

**Scheme 2:**
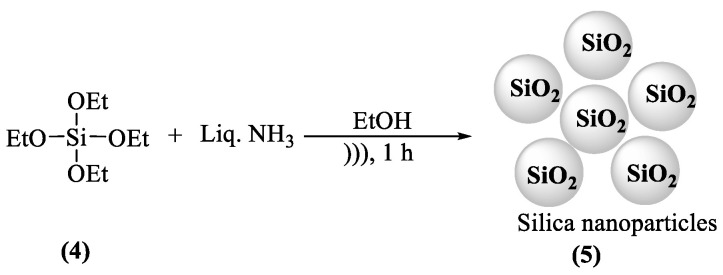
Synthesis of silica nano-particles **(5)** (presenting with animated silica).

Silica nanoparticles were synthesized by hydrolysis of the tetraethoxysilane (TEOS) **(4)** using modified St$\ddot{o}$ber process [41,42]. In a 100 mL of conical flask, 1.00 mL of TEOS **(4)** was added to 10.00 mL of absolute alcohol and the reaction was sonicated for 5 min. Further, 10.00 mL of 25% ammonium hydroxide solution (NH_4_OH) and 10.00 mL of absolute alcohol were added slowly to the reaction mixture while sonication. The reaction mixture was allowed to sonicate for 1 h to obtain white turbid suspension, which was further centrifuged for 2 h. The separated nano-composites of silica were washed with water and re-dispersed in alcohol to centrifuge again for 1 h. Finally, powdered silica **(5),** so obtained after repeated filtrations was dried in vacuum oven and calcinated at 400 °C in a furnace (Scheme 2).

**Scheme 3:**
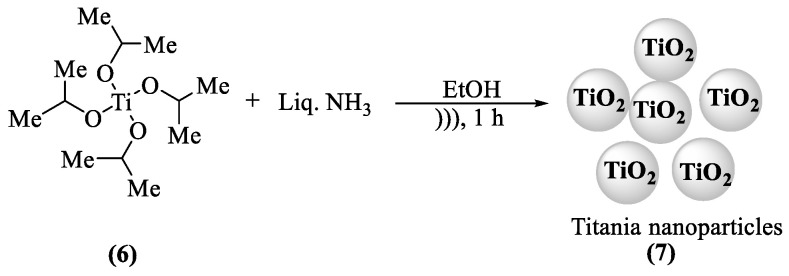
Synthesis of titanianano-particles **(7)** (presenting with animated titania).

Likewise, using similar approach, synthesis of the titania **(7)** was also carried out by taking tetraisopropylorthotitanate (TIPT) **(6)** as precursor (Scheme 3) and nano-composites were obtained in good amount.

Further, to create the organic molecule holding sites these synthesisednano-composites **(5)** and **(7)** were functionalized with 3-aminopropyl triethoxysilane, which provided binding sites to the organic chemosensor. 

### 2.3. Functionalization of SiO_2_ and TiO_2_ with 3-Aminopropyl Triethoxysilane (APTES)

3-Aminopropylated silica nano-composites APTES@SiO_2_ **(9)** were synthesized by adding 2.00 mL of 3-aminopropyl triethoxysilane (APTES) **(8)** to 50.00 mL of vigorously stirred dispersion of synthesized silica nano-particles **(5)** in absolute alcohol and the resulting mixture was allowed to stir overnight at room temperature. These freshly synthesized APTES@SiO_2_ nano-composites were purified by centrifugation and re-dispersion in alcoholic solution. In order to remove unreacted 3-aminopropyl triethoxysilane and to get analytical pure nano-composites **(9)**, the process was repeated thrice [43].

**Scheme 4:**
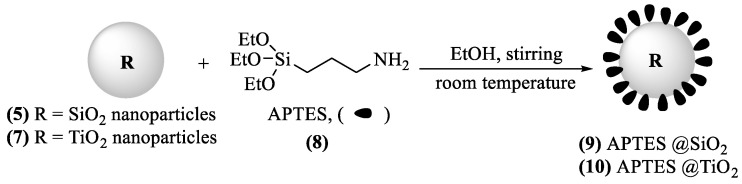
Synthesis of functionalized nano-particles of silica **(9)** and titania nano-particles **(10)**.

Similarly, synthesis of the APTES@TiO_2_ **(10)** was also carried out by using above procedure with titania**(7)** as a core material (Scheme 4). APTES@TiO_2_ was also furnished in good amount.

### 2.4. Synthesis of the Organic-Inorganic Nano-Composites ***(11, 12)*** of Chemosensor ***(3)***

**Scheme 5:**
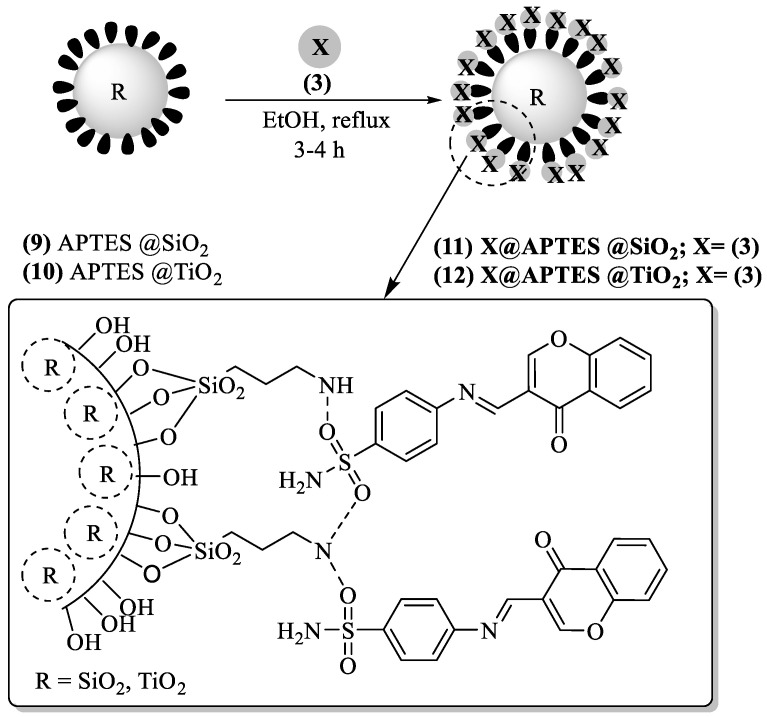
Synthesis of organic-inorganic nano-composites of silica **(11)** and titania **(12)**.

Finally, after functionalization of the nano-particles **(5)** and **(7)**, to obtain adhering sites for metal ions over **(9)** and **(10)**, organic chemosensor **(3)** was coated over their surface to form intact organic-inorganic nano-composite materials **(11)** and **(12)**. These were further used for sensing analysis as such without any alterations. Immobilization method was used to obtain the desired organic–inorganic nano-composites [43]. The synthesized chemosensor **(3)** was immobilized on APTES@SiO_2_ **(9)** and APTES@TiO_2_ **(10)**. The mixture of APTES@SiO_2_ **(11)** was refluxed with chemosensor **(3)** for 3–4 h in 15.00 mL acetone to obtain raw nano-composites of silica. The product so obtained was washed with acetone (2 × 40.00 mL) twice and dried in a vacuum oven to afford organic-inorganic nano-composites **(11)** as an analytically pure sensing material (Scheme 5).

Likewise, APTES@TiO_2_ **(12)** was refluxed with chemosensor **(3)** for 3–4 h in 15.00 mL acetone to obtain raw nano-composites of titania. Washing with acetone (2 × 40.00 mL) and drying in vacuum oven furnished organic–inorganic nano-composites **(12)** as a final sensing material (Scheme 5).

## 3. Results and Discussion

### 3.1. Chemoreceptor Spectral Studies 

The photo-physical behavior of the nano-composites **(11)** and **(12)** was tested independently followed by study of variation obtained in emission analysis of nano-composites **(11)** and **(12)** in the presence of various metal ions and anions. Initially, the emission spectra of the nano-composites **(11)** and **(12)** of 10 ppm concentration were analyzed before and after addition of metal ions and anions with excitation at 290 nm. The result indicated that the specific variations in emission intensity of **(11)** was obtained with Hg^2+^ ions only and no other metal ion was able to alter the emission peak of the nano-composites **(11)**. Additionally, linear fitting equation between the fluorescence intensity of nano-composite **(11)** and Hg^2+^ ion concentration (by varying concentration from 4–50 nM) was applied to verify the emission response of **(11)** with Hg^2+^ ion. Similarly, emission spectral analysis of nano-composite **(12)** at same concentration was recorded by adding various metal ions and anions that also showed selectivity towards Hg^2+^ ions. Molar increment experiment of Hg^2+^ ion was conducted between 2–35 nM concentrations as per the range of detection (Section 3.2, Section 3.3 and Section 3.4).

### 3.2. Chemistry of Nano-Composites ***(11)*** and ***(12)*** and Their Turn-On Emission Due to Hg^2+^ Ions

The structures of nano-composites **(11)** and **(12)** were designed by keeping in mind the need of heteroatomic sites for binding of the analytes; that can adhere either because of its specific size or due to atom selective coordination linkage [44]. Further, nanoparticles provided solid phase for attachment which can hold it for long period and had strong binding with the surface.

**Figure 1:**
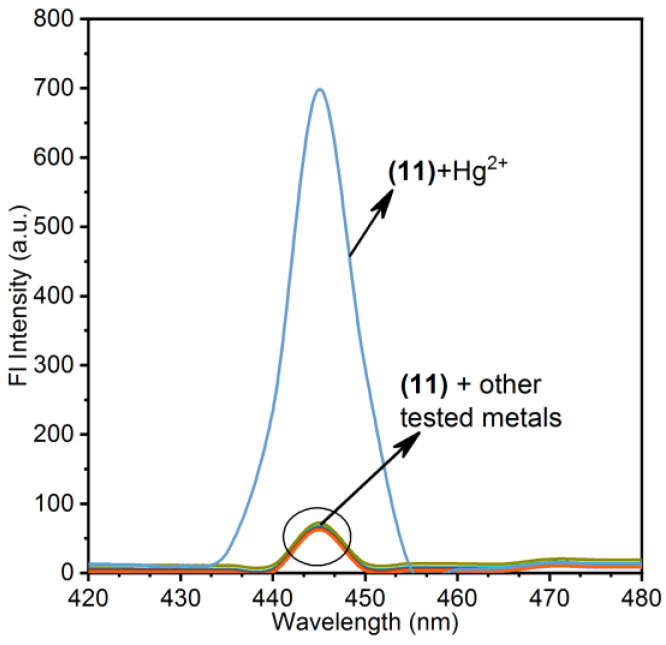
Fluorescence response of **(11)** (10 ppm) towards various cations at excitation of 290 nm.

Prior to investigation of Hg^2+^ ion selective emission studies of **(11)** (10 ppm), emission profile of **(11)** was recorded as a free sensor with excitation wavelength of 290 nm. Fluorescence emission data revealed that the nano-composites **(11)** exhibited a distinct peak at 445 nm (blue emission) with very low intensity corresponding to excitation at 290 nm and envisaged that nano-composites **(11)** were not fluorogenic in nature. Further, nano-composites **(11)** (10 ppm) were tested against various metal ions (Al^3+^, Ag^+^, Ba^2+^, Ca^2+^, Cd^2+^, Cu^2+^, Cr^3+^, Co^2+^, Fe^3+^, Hg^2+^, K^+^, Li^+^, Mn^2+^, Mg^2+^, Na^+^, Ni^2+^, Pb^2+^ and Zn^2+^), merely Hg^2+^ showed an unambiguous intensity growth (Figure 1). This increment in intensity is considered as one of the relevant and valid ways to check specific ion presence as turn-on fluorescence. Auspiciously, fluorescent enhancement factor (FEF) was found to be 10.9 times hiked in intensity of peak at 445 nm from fluorescence plot of **(11)** and Hg^2+^ ions in aqueous medium.

**Figure 2:**
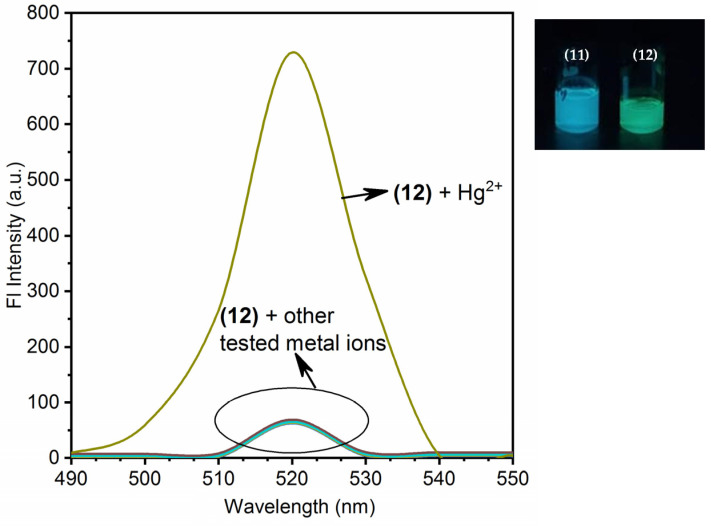
Fluorescence response of **(12)** (10 ppm) towards various cations at excitation of 290 nm (Inset: image of nano-composite **(11)** and **(12)** on addition of Hg^2+^ ions).

Whereas, emission profile of nano-composites **(12)** was also recorded as a free sensor with excitation wavelength of 290 nm. Emission spectral analysis of the nano-composites **(12)** exhibited a distinct peak at 520 nm (green emission) corresponding to excitation at 290 nm with negligible intensity of 50.00 a.u. Additionally, nano-composites **(12)** (10 ppm) were tested against various metal ions and herein, again, only Hg^2+^ ion showed emission intensity enhancement (Figure 2). This also shifted the sensor emission to the fluorescence turn-on mode for the toxic Hg^2+^ ions. In case of titania coated ligand **(12)**, FEF was found to be 11.1 times hiked in intensity of peak at 520 nm from fluorescence plot of **(12)** and Hg^2+^ ions in aqueous medium, which was found to be more than the silica based nano-composites **(11)**. 

**Figure 3:**
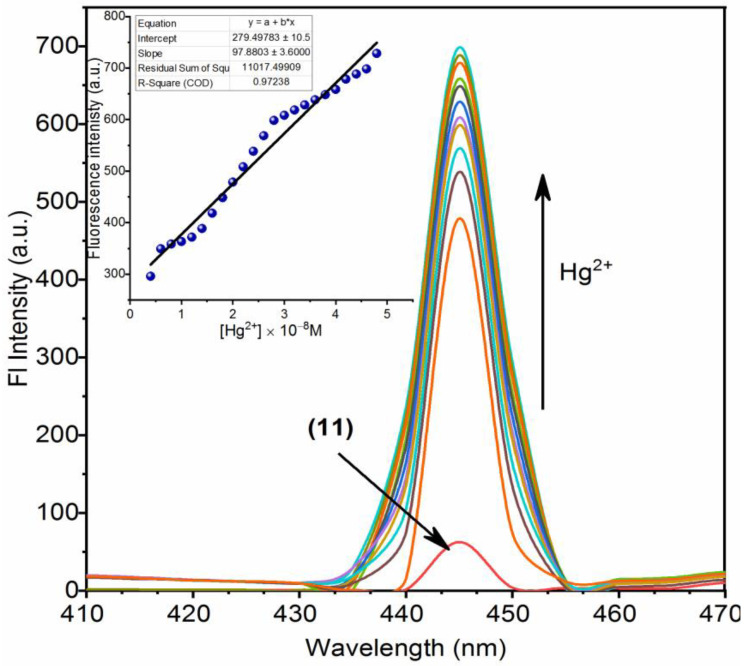
Emission spectra of the sequential addition of Hg^2+^ ions (4 nM–50 nM) in 10 ppm of nano-composite **(11)**; inset linear fitting pot to calculate LOD and LOQ @445 nm.

The fluorescence titration profile is shown in Figure 3. The intensity of the peak at 445 nm of **(11)** gradually increased with increase in the concentration of the Hg^2+^ ions without any shift in band emission wavelength. By applying the linear fitting to the plot of Hg^2+^ ions concentration and intensity change, the values of LOD and limit of quantification (LOQ) were assessed with the help of equation LOD = 3S_d_/slop and LOQ = 10S_d_/slop, where S_d_ is standard deviation. The values of LOD and LOQ were found to be 41.2 nM and 137.3 nM (R^2^ = 0.972), respectively (Figure 3).

**Figure 4:**
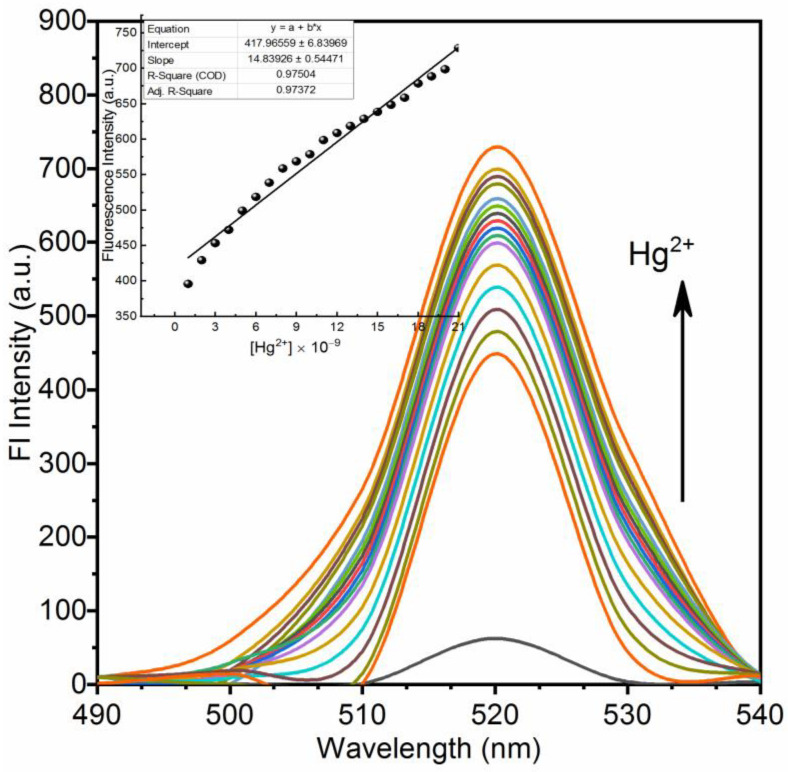
Emission spectra of the sequential addition of Hg^2+^ ions (1–20 nM) in 10 ppm of nano-composite **(12)**; inset linear fitting plot to calculate LOD and LOQ @520 nm.

Further, molar increment titration experiment was conducted to study the binding interactions between **(12)** and Hg^2+^ ion. The gradual increase in the weak emissive band of **(12)** at 520 nm was obtained with increase in concentration of Hg^2+^ ions (upto 20 nM) (Figure 4) and the complex **[(12)+Hg^2+^]** became fluorogenic in nature. From the previous demonstration, we observed that the emission intensity of **(12)** is directly proportional to the addition of the Hg^2+^ ions concentration. Further, the LOD was calculated by linear emission fitting for **[(12)**+**Hg^2+^]** and was found to be 18.8 nM, followed by LOQ of 62.83 nM (R^2^ = 0.975) (Figure 4 inset). These values of LOD in aqueous medium for the detection of toxic Hg^2+^ with silica and titania were found to be very low in the terms of aqueous medium analysis comparative to the some recently reported solid phase dispersive nano-composite chemosensors (Table 1). From the Table 1, it can be depicted that few sensors are available for the Hg^2+^ detection below 20 nM concentration with fluorescence spectroscopy technique and the sensors for detection limit less than 10 nM concentration were dependent on potentiometric and digital information method, and were used for catalytic activities rather than sensing behavior.

### 3.3. Anion Sensing Analysis of Nano-Composites ***(11)*** and ***(12)***

**Figure 5:**
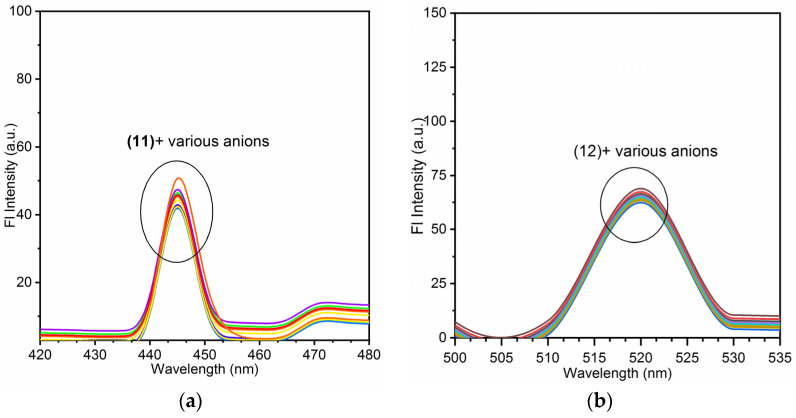
Emission spectra of nano-composites (**a**)→**(11)**; (**b**)→**(12)** in the presence of various anions.

To check the efficacy of nano-composites **(11)** and **(12)** against various anions, the emission spectra of **(11)** and **(12)** were recorded with addition of 50 ppm of various anions *viz.* F^−^, I^−^, CN^−^, Cl^−^, Br^−^, SO_4_^2−^, HCO_3_^−^, OH^−^, C_2_O_4_^2−^, HSO_4_^2−^, SO_3_^2−^, and NO_2_^−^. It was observed that the addition of different anions did not alter the emission spectra either of two nano-composites, as indicated from Figure 5a,b. After analysing the sensing ability of nano-composites **(11)** and **(12)**, further interference study was conducted to check the potential behavior of the sensors in the presence of obstructive environment. 

### 3.4. Competitive Binding Analysis (Interference Analysis)

**Figure 6:**
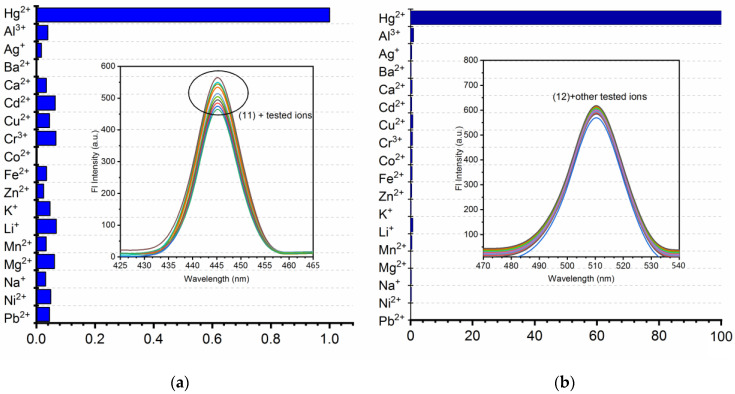
Normalized plots of nano-composites (**a**)→**(11)**; (**b**)→**(12)** in the presence of various tested metal ions; inset emission spectra of nano-composites (**a**)→**(11)**; (**b**)→**(12)** with different tested ions.

Competitive binding analysis was carried out to calculate the realistic value of **(11)** as an Hg^2+^ ion selective chemosensor in the rival environment of the intrusive metal ions in aqueous medium. The experiment was carried out by taking 10 ppm of the nano-composites **(11)** and 15 ppb of Hg^2+^ ions in deionised water and was spiked with all tested metal ions and anions. No visual alterations in the emission intensities were recorded in the spiked samples of the metal ions (Figure 6a inset). Therefore, it can be implicated that **(11)** exhibited high sensitivity, selectivity and turn-on fluorescence response towards Hg^2+^ ions. Additionally, to check the efficacy of **(11)**, normalized data plot of Hg^2+^ ions along with various intruding metal ions in presented in Figure 6a.

Additionally, the selectivity of the nano-composites **(12)** towards Hg^2+^ ions was tested with addition of 15 ppb of other interfering ions. It can be seen from the experimental data that the addition of various ions had no or negligible effect on the emission intensity of **[(12)+Hg^2+^]** complex (Figure 6b inset). The normalized plot of **[(12)+Hg^2+^]** was plotted to compare the emissive behavior of the **[(12)+Hg^2+^]** complex with other studied metal ions (Figure 6b). These results proved that nano-composites **(11)** and **(12)** are promising emissive sensors for toxic Hg^2+^ ions in aqueous samples even in the presence of most intruding metal ions and anions. 

### 3.5. Plausible Mechanism of Sensing of ***(11)*** and ***(12)***

**Scheme 6:**
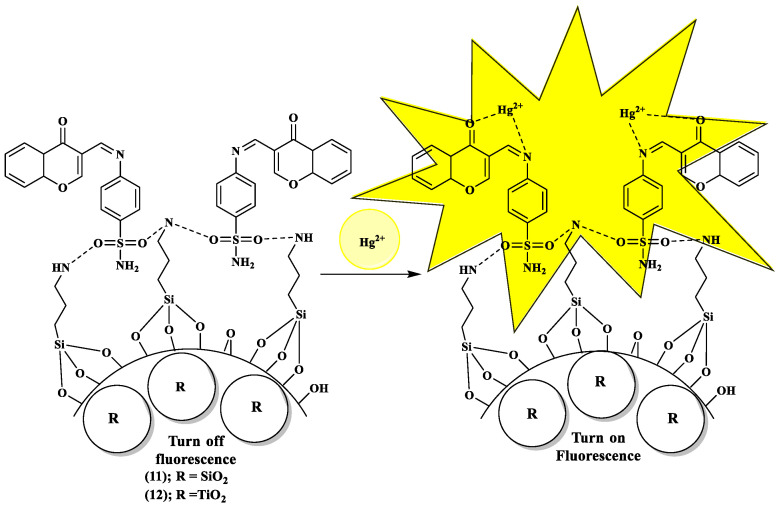
Plausible mechanism of Hg^2+^ sensing via Nano-composite **(11)** and **(12)**.

From the above data, it was observed that various factors can be listed to rationalize the observed emission enhancement of nano-composite **(11)** and **(12)** by Hg^2+^ (Scheme 6). The weak fluorescence of the **(11)** and **(12)** in the absence of Hg^2+^ can be attributed due to instant cis-trans isomerization across the imine (C=N) bond. The possible binding mechanism of **(11)** and **(12)** with Hg^2+^ that led to the fluorescence changes is shown in Scheme 6. Nano composites were most likely to bind with Hg^2+^ ions through the corresponding oxygen and nitrogen atoms which results in fluorescence enhancement due to the ligand to metal charge transfer [L-MCT] nature of Hg^2+^ Upon chelation of probe with Hg^2+^ Chelation Enhanced Fluorescence (CHEF) produced within the organic moiety of the nano-composites **(11)** and **(12)** [57]. It was also found that the detection limit of Hg^2+^ is lesser in case of titania as compared to the silica coated organic ligand attributed to the fact that the size and the amount of organic ligand coated on the surface of TiO_2_ is more than that of the SiO_2_, which was also studied in the Solid state NMR and BET studies mentioned ahead.

### 3.6. Effect of pH on Nano-Composites ***(11)*** and ***(12)*** with Hg^2+^ Ions

**Figure 7:**
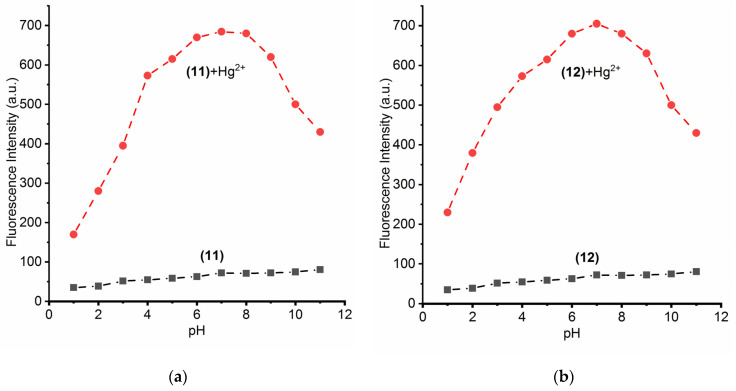
Effect of pH on nano-composites (**a**)→**(11)** and **[(11)+Hg^2+^]**; (**b**)→nano-composites **(12)** and its complex **[(12)+Hg^2+^]**.

pH of the solution is an important variable in sensing studies of aqueous samples as it affects the surface of the nano-composites and coordination sites while sensing of analytes. Therefore, the effect of pH on the sensing analysis of nano-composites **(11)** and **(12)** were carried out in the range of pH 2–11. Various solutions of nano-composites **(11)** and **(12)** over a wide range of pH were prepared along with the different solutions of complexes **[(11)+Hg^2+^]** and **[(12)+Hg^2+^]** to compare their sensing behavioral changes. 0.1 N NaOH and 0.1 N HCl were utilized to adjust the pH of the different solutions of nano-composites **(11)** and **(12)**. As shown in the Figure 7a,b; pH values from 1–3 (acidic) affected the Hg^2+^ ion complexes with respective nano-composites **(11)** and **(12)**. This is attributed to the blocking of the coordination sites of nano-composites **(11)** and **(12)** with excessive H^+^ ions, which in turn, reduced the ligand to metal charge transfer (L-MCT) and was responsible for decrease in emissive intensity of respective complexes with Hg^2+^ ions. Furthermore, with increase in pH up to neutral value i.e., pH 4–10, it was visualized that coordination sites became freely available for binding of Hg^2+^ ions, which again enhanced the intensity of the nano-composites **(11)** and **(12)** to the initial level. Proceeding towards the alkaline pH due to excess of hydroxide ions in the solutions, tendency of Hg^2+^ ions towards OH^−^ increased due to opposite charges and hence resulted the formation of Hg(OH)_2_. According to literature reports, Hg(OH)_2_ is highly unstable and is converted to HgO in aqueous media readily [58]. This was found to be the fundamental reason behind quenching of the emission intensity of complexes **(11)** and **(12)**. Thus, the results indicated that the optimum pH for the detection analysis of toxic Hg^2+^ ions is 4 to 10 at room temperature.

### 3.7. Characterization of Nano-Composites ***(11)*** and ***(12)***

#### 3.7.1. FT-IR Studies 

Structural analysis of the nano-composites **(11)** and **(12)** was performed to rule out the surface modifications of synthesized SiO_2_ and TiO_2_ with 3-aminopropyl triethoxysilane (APTES) **(8)** and organic ligand **(3)**. 

**Figure 8:**
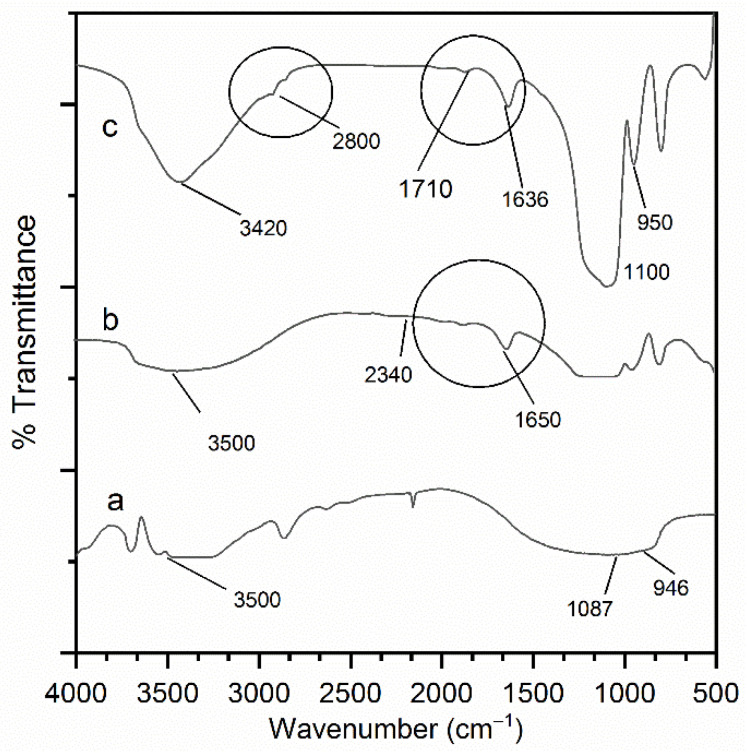
Comparative FT-IR spectra of the (**a**) SiO_2_ (**b**) APTES@SiO_2_ and (**c**) **(3)**@APTES@SiO_2_.

FT-IR spectrum of the SiO_2_ showed the presence of functional groups such as Si–O, Si–O–Si and Si–OH (Figure 8). The IR bands centred at 1087 and 946 cm^−1^ were assigned to asymmetric stretching of the Si–O–Si and Si–OH groups respectively. Whereas the symmetric stretching vibration and bending vibration bands of Si–O–Si were clearly present at 790 and 480 cm^−1^, respectively. Additionally, the spectrum presented a broad band centred at 3500 cm^−1^ due to hydroxyl (O-H) group of silanol (Si–OH) moiety and physisorbed water. However, the FT-IR spectrum of APTES@SiO_2_ (b) showed a significant decrease in the silanol and hydroxyl group intensities due to the reaction between surface hydroxyl group and ethoxy (O–C_2_H_5_) group of APTES **(8)** during surface modification reaction. The functionalization of surface of silica was confirmed by appearance of new weak bands at 2324.3 and 2340 cm^−1^, which were assigned to methylene groups (–CH_2_–) and band around 1650 cm^−1^ attributed to NH_2_ group of APTES **(8)**. Further, the FT-IR spectrum of nano-composites **(11)** (c) showed the medium band centered at 1636 cm^−1^ due to stretching vibrations of imine (–C=N–) linkage and strong vibration band at 1100 cm^−1^ which was attributed to sulfonamide (S=O) group. The Si–O–Si group vibrations in nano-composites **(11)** were shifted to 950 and 476 cm^−1^ from 946 and 480 cm^−1^, respectively due to surface modifications. Thus, change in the shifting of silica bands and appearance of new imine and sulfonamide vibrational bands confirmed the successful adheration of organic ligand **(3)** to the surface of silica to form nano-composites **(11)** [59]. 

**Figure 9:**
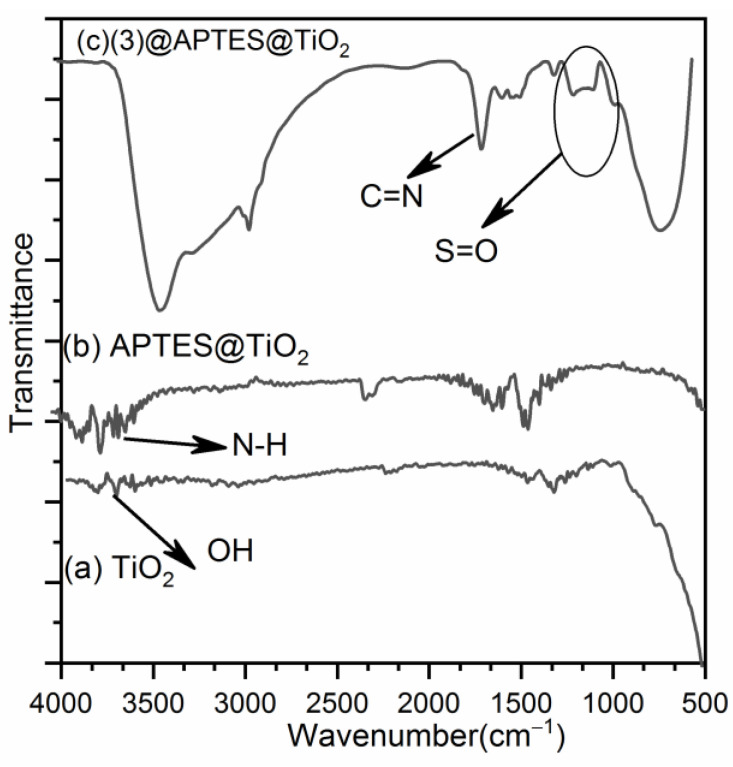
Comparative IR spectra of the (**a**) TiO_2_, (**b**) APTES@TiO_2_ and (**c**) **(3)**@APTES@TiO_2_.

Similarly, the FT-IR spectral studies were also conducted to verify the insertion of organic moieties into the nano-composites **(12)**. Figure 9 showed the FT-IR spectra of TiO_2_, APTES@TiO_2_ and **(3)**@APTES@TiO_2_. In all samples, the characteristic bands of titania framework at around 800 cm^−1^ (symmetric stretching vibrations of Ti–O), 960 cm^−1^ (symmetric stretching vibration of Ti–OH), 1200 cm^−1^ (asymmetric stretching vibrations of Ti–O–Ti), and 3400 cm^−1^ (physisorbed water molecules) and 3437 cm^−1^ (stretching vibrations of OH groups) were present. The new bands within the range of 2409–2486 cm^−1^ are characteristic of aliphatic alkyl-chain C–H vibrations (Figure 9b). In Figure 9c, new bands at around 1179 and 1454 cm^−1^ were assigned to the S=O and C=N stretching vibrations of imine linkage. The other observed band around 1538 cm^−1^ assigned to –NH vibrations. The band at 1652 cm^−1^ was assigned to the imine bond formed which confirmed the coating of functionalized nanoparticles **(7)**.Thus, the FT-IR spectra of titania **(7)** and functionalized titania **(12)** also confirmed the incorporation of the fluorophore groups in the TiO_2_ framework.

#### 3.7.2. Solid-State ^13^C CPMAS and ^29^Si CPMAS NMR Spectroscopy

**Figure 10:**
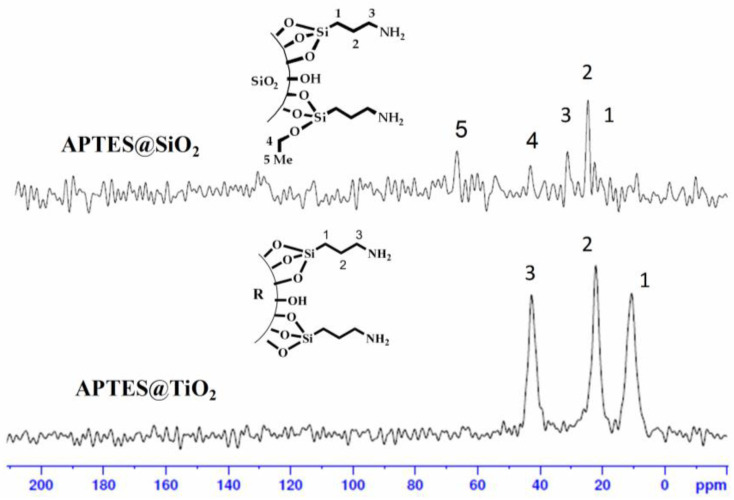
Solid State ^13^C CPMAS NMR spectra of APTES@SiO_2_ and APTES@TiO_2_.

The fuctionalization of silica and titania nanospheres with APTES and the organic compound **(3)** was investigated with ^13^C and ^29^Si CPMAS NMR spectroscopy studies. In the ^13^C spectra of APTES@SiO_2_, three resonance peaks appeared around δ 22.18, 24.31 amd 30.83 ppm, which were assigned to three carbon atoms of the integrated 3-aminopropyl chain of APTES. Similarly the ^13^C CPMAS NMR spectrum of APTES@TiO_2_ also showed three peaks corresponding to δ = 10.57, 22.07 and 42.67 ppm, which were also assigned to the incorporated aminopropyl chain. But in the ^13^C spectrum of the APTES@SiO_2_, two low intensity additional peaks were also seen at δ 66.15 ppm and 42.69 ppm indicating the existance of tiny amounts of unreacted ethoxy group of APTES as shown in Figure 10. The above data showed that as compared to silica, titania surface was more effectively covered and strongly bind to APTES that in turn also helped the organic compound **(3)** to adhere the nano-composite surface more efficiently.

**Figure 11:**
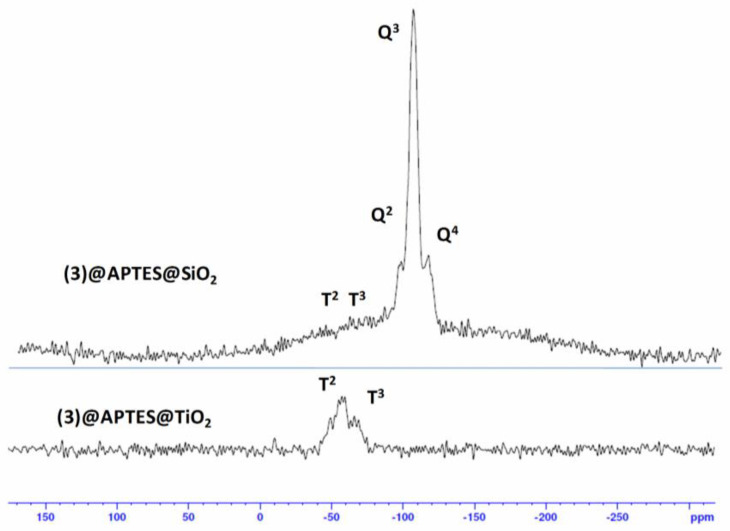
Solid-state ^29^Si CPMAS NMR spectra of **(3)**@APTES@SiO_2_ and **(3)**@APTES@TiO_2_.

The solid-state ^29^Si CPMAS NMR spectra of **(3)@**APTES@SiO_2_ and **(3)@**APTES@TiO_2_ are shown in Figure 11. The peaks appeared around −52.21ppm and −57.32 ppm corresponded to silanol group of the C–Si(OSi)_2_(OH) group (T_2_) and the C–Si(OSi)_3_ group (T_3_), respectively, provided clear evidence that the nano-composite sensing material **(3)@**APTES@SiO_2_**(11)** was made up of a silica scaffold with an organic group covalently bonded to SiO_2_ nanoparticles. In addition, the spectrum showed additional peaks that associated to silica’s inorganic polymeric structure: Si(OSi)_4_(3D) group (Q_4_) was allocated −111.49 ppm and 113.63 ppm, while the free silanol group of Si(OSi)_3_OH was assigned to 101.4 ppm (Q_3_) and linear C-Si(O-Si)_3_ to −91.34 ppm (Q_2_). Also the ^29^Si NMR of the **(3)@**APTES@TiO_2_**(12)** showed peaks around δ 49.32 and 55.10 ppm (T_2_) corresponding to Ti-O-Si-C linkage which confirmed the covalent linkage of organic motiey. Further, other peaks around 59.33, 63.50, and 68.93 ppm (T_3_) corresponded to the SiO_2_ scaffold [60].

#### 3.7.3. Elemental (C, H, N) and Surface Area Analysis (BET Studies)

Elemental studies for the determination of C, N and H percentages were conducted to verify the successful modifications of nanoparticles **(5)** and **(7)** with organic ligand **(3)**. The presence of the appropriate percentage of ‘C’ and ‘N’ in **(9)**, **(10)**, **(11)** and **(12)** materials confirmed the formation of organic ligand **(3)** coated nano-composites **(11)** and **(12)** as shown in Table 2. To ensure the surface modifications with organic ligand, surface studies of the prepared materials **(9)**, **(10)**, **(11)** and **(12)** were conducted. In the BET studies, the surface area of the nano-composites **(11)** and **(12)** were compared with bare silica nanoparticles **(5)** and bare titania nano-particles **(7)**. 

As listed in Table 2, the BET surface area of the silica and functionalized materials were found to be 201.81 m^2^g^−1^ and 113.21 m^2^g^−1^, respectively which were further decreased to 77.56 m^2^g^−1^ for nano-composites **(11)**. As expected, the BET studies revealed that the surface area of the nanoparticles decreased in order of SiO_2_**(5)** > APTES@SiO_2_**(9)** > **(3)**@APTES@SiO_2_**(11)** nano-composites, which confirmed the modification of the silica surface. It was seen that the immobilization of nano-particles SiO_2_**(5)** with the ligand **(3)** and APTES blocked nitrogen assess onto the surface of **(5)**. These results were in good agreement with the previous studies.

Also the surface area of titania compared to APTES coated titania and organic moiety **(3)** coated titania decreased with every succeeded coating on titania as shown in Table 2 and confirmed the formation of intact nano-composites **(12)**. Comparison of BET data of nano-composites **(11)** and **(12)**, revealed that nano-composites **(12)** were more intensely coated with the organic ligand **(3)** than to nano-composites **(11)** by reacting same amount of organic ligand **(3)**, and was responsible for low detection limit of Hg^2+^ ions with **(12)** than **(11)**. 

#### 3.7.4. Field Emission Stimulated Electron Emission (FE SEM) and Energy Dispersive X-ray Analysis (EDX)

**Figure 12:**
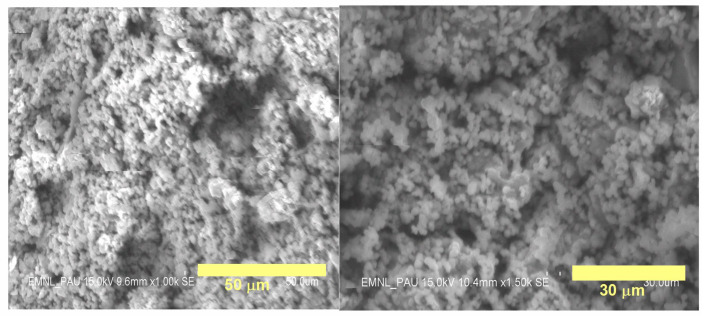
FE SEM images of functionalized organic-inorganic nano composites **(11)**.

The size, morphology and topographical studies of synthetic nanoparticles were examined using a Field Emission Stimulated Electron Emission (FE SEM). Figure 12 showed the FE SEM images of nano-hybrid sensing material **(11)**. FE SEM micrographs revealed that nanoparticles were spherical in shape having rough coating of organic ligand over their surface and were not covalently attached all over, which was maintained throughout and much of particles did not agglomerate into clusters. It was also found that the average particle size of functionalized SiO_2_ nanoparticles was approximately 300 nm.

**Figure 13:**
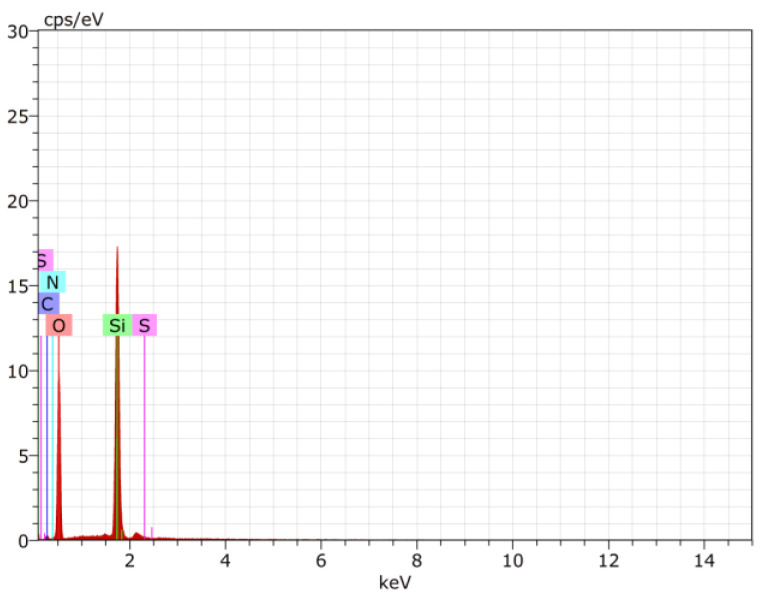
EDX of the of functionalized organic-inorganic nano composites **(11)**.

Energy Dispersive X-ray studies (EDX) revealed the presence of sulfur and carbon which confirmed the coating and functionalization of the nano-composites **(11)**. The percentage of all the elements present in **(11)** depicted that oxygen was present in highest amount followed by the silicon which form the core of nano-composites **(11)**. Presence of carbon and sulfur confirmed the coating of the organic ligand **(3)** over APTES modified nano-particles **(9)** (Figure 13, Table S1).

**Figure 14:**
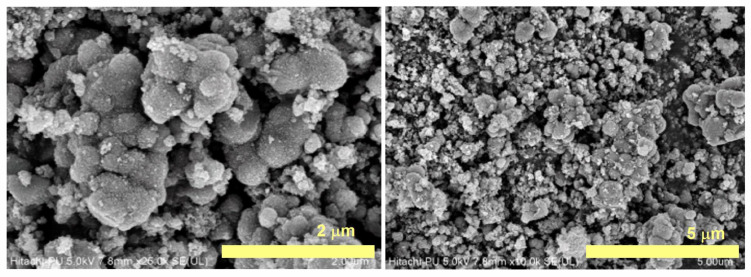
FE SEM images of functionalized organic-inorganic nano composite **(12)**.

**Figure 15:**
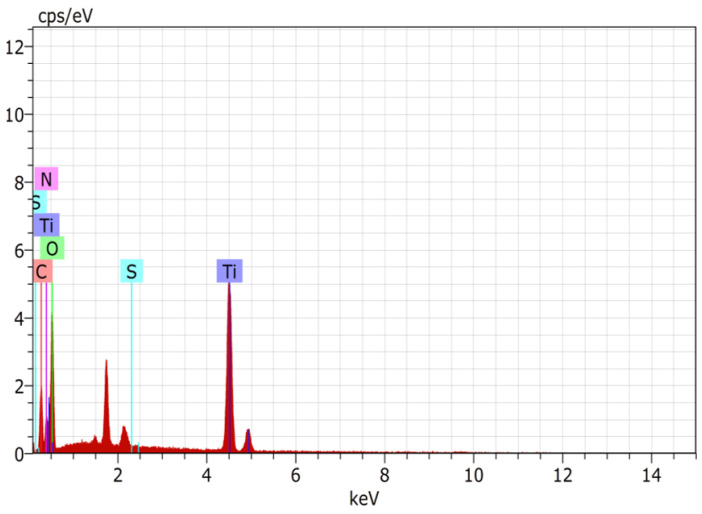
EDX of the functionalized organic-inorganic nano composites **(12)**.

The FE SEM micrographs of **(12)** was recorded and it showed that the nano-composites **(12)** possessed rough coating of organic ligand over its surface and was not covalently attached all over uniformly and the average particle size of functionalized TiO_2_ nano-composite was also approximately 210 nm. Additionally, the presence of sulfur and carbon in EDX spectra of **(12)** confirmed the coating of organic ligand **(3)** on functionalized nanoparticles **(10)** to finally obtain nano-composites **(12)**. The percentage of all the elements present in **(12)** depicted that oxygen was present in highest amount followed by the titanium which formed the core of nano-composites **(12)** (Figure 14 and Figure 15; Table S2).

#### 3.7.5. Transmission Electron Microscopy (TEM) Analysis

**Figure 16:**
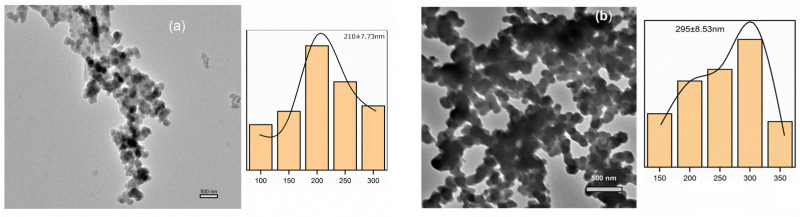
TEM images of functionalized organic-inorganic nano composite (**a**)—**(11)** and (**b**)—**(12)**.

Tem studies were also conducted to check the size and morphology of the synthesized nano-composites **(11)** and **(12)** by using field emission gun at voltage of 300 kV. The samples were prepared by suspending nano-composites in absolute alcohol and then by drying a drop of the same on carbon coated copper TEM grid. TEM micrographs revealed that nano-composites **(11)** and **(12)** were mostly mono-dispersive and with an average size of 210 ± 7.73 and 295 ± 8.82 nm of nano-composite **(11)** and **(12)**, respectively (Figure 16).

#### 3.7.6. X-ray Diffraction (XRD) Studies

**Figure 17:**
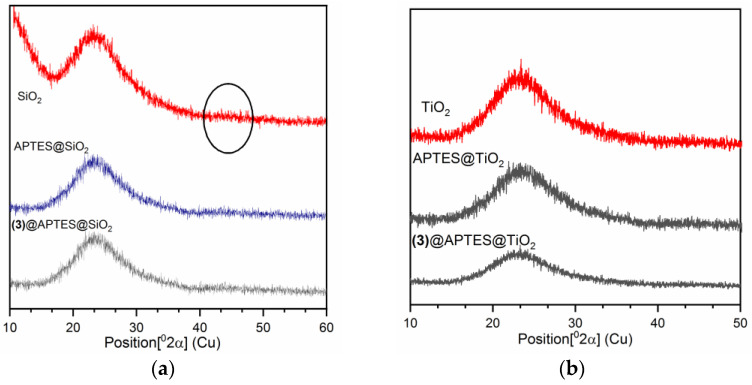
XRD pattern of (**a**)→SiO_2_, APTES@SiO_2_ and **(3)**@APTES@SiO_2_**(11)**; (**b**)→TiO_2_, APTES@TiO_2_ and **(3)**@APTES@TiO_2_.

X-ray powder diffraction studies were conducted to confirm the structural characterization of SiO_2_**(5)**, APTES@SiO_2_**(9)** and **(3)**@APTES@SiO_2_nano-composites **(11)**. Powder XRD pattern was obtained from Bragg’s equation λ = 2dsinθ using CuKα radiations, as shown in Figure 17a. An amorphous peak with an equivalent Bragg’s angle appeared at 2θ = 23°, corresponding to the SiO_2_ prepared by modified Stöber method after thermal treatment at 400 °C temperature. The single broad halo is due to average molecular separation in the amorphous phase and it confirmed the non-crystalline nature of silica prepared by modified Stöber’s method. The literature citation revealed that 2θ value of amorphous silica depends upon temperature treatment and water to tetraethoxysilane (TEOS) ratio. Our measured 2θ values of silica were found to analogous to the reported 2θ value of silica [61]. Further, the XRD studies of the APTES functionalized silica **(9)** and nano-composites **(11)** showed 2θ at 23° only with increase in peak intensities confirmed the immobilization of APTES and organic ligand **(3)** on silica surface with confined amorphous character of silica.

Similarly, structural characterization of TiO_2_, APTES@TiO_2_ and **(3)**@APTES@TiO_2_**(12)**, was carried out with powder XRD analysis and the resulting patterns are presented in Figure 17b. All samples showed a single broad peak indicating their amorphous nature and preserved the non-crystalline nature even after functionalization with APTES and organic moieties, which implied that TiO_2_ nanoparticles were stable enough to experience the chemical modification reactions same as that of silica nano-composites **(11)**. However, XRD peak intensities decreased on moving from TiO_2_ to APTES@TiO_2_ to **(3)**@APTES@TiO_2_ **(12)** which also indicated the successive immobilization of APTES and **(3)** onto the TiO_2_ matrix.

On comparing, the powder XRD data of nano-composites **(11)** and **(12)**, it was found that there was more effective coating of ligand **(3)** over the APTES@TiO_2_ surface than APTES@SiO_2_ surface. Additionally, more decrease in peak intensity was noticed on moving from APTES@TiO_2_ to **(3)**@APTES@TiO_2_**(12)** comparative to the fall in peak intensity from APTES@SiO_2_ to **(3)**@APTES@SiO_2_**(11)** (2θ = 23°) and pointed to the lower limit of detection with nano-composites **(12)**.

### 3.8. Application on Real Samples 

To authenticate the practical applicability of the nano-composites **(11)** and **(12)**, the composites were also applied to the mercury determination in real samples. Nano-composites **(11)** and **(12)** were successfully applied in three different types of water (tap, distilled and bottled water) for the detection of Hg^2+^ ions. Tap water was filtered through Whatman filter paper prior to its use. After dispersing **(11)** and **(12)** in each sample, the fluorescence spectra of the prepared samples were recorded thrice. Further, the samples were spiked with known amounts of Hg^2+^ ions solution and their emissions intensities were analyzed. From the respective calibration curves of **[(11)+Hg^2+^]** and **[(12)+Hg^2+^]** complexes, concentrations of Hg^2+^ ions were determined in spiked samples. The results given in Table 3 indicated that there was a good agreement between the spiked and measured number of ions. It was observed that recovery percentages for the known amount of spiked Hg^2+^ ions were found between 98–100%, which made the present approach authentic and reliable for real sample assessment.

## 4. Conclusions

In conclusion, we have successfully synthesized and characterized nano-composites **(11)** and **(12)** of silica and titania (non-fluorogenic), which were evaluated as optical sensors/fluorescence turn-on sensors for Hg^2+^ ions. It was found that the particle size of nano-composites **(12)** were lesser than that of **(11)**. Pleasantly, only Hg^2+^ ion induced the metal-ligand chelation enhanced fluorescence in both nano-composites, while all other ions showed negligible response. None of the intruding ion altered the sensitivity and selectivity of nano-composites towards Hg^2+^ ions.The detection limits of the **(11)** and **(12)** were found to be 41.2 nM and 18.8 nM, respectively. Data obtained from p-XRD, BET and EDX studies showed that there were more amount of ligand **(3)** adhered on the nano-composite **(12)** in comparison to **(11)**, which was found to be the plausible reason behind the lower detection limit of **(12)**. In addition, the present emission based analytical method provided an economic and simple synthetic route for a selective and a sensitive quantification of the one of the toxic metal Hg^2+^ ionin environmental samples.

## Figures and Tables

**Table 1:** Comparative examples of some of recently reported sensors of Hg^2+^ ions.

Ligand	LOD	Limitations	Reference
Solid phase peptide	7.59 nM	Tedious reaction procedure	[45]
Azobenzene	50 µM	Tedious reaction procedure	[46]
Anthracen-9(-ylmethylene)hydrazine	220 nM	pH specificity, Effective over pH 5–8 only	[47]
Merocyanin dye	0.27 µM	Molecule disrupted by UV light	[48]
Pyrazole based derivative	0.473 µM	Detection limited to pH 6–11	[49]
2-Hydroxy-5(-4-nitrophenyl) diazonyl benzaldehyde	6.1 µM	Solvent specificity, ligand worked in presence of DMSO only	[50]
3-(4-Dimethylamino-phenyl) allylidene-3-nitro benzohydrazide	0.11 µM	pH specificity, effective pH > 7 only	[51]
8-Hydroxy-7-(4-nbutyl phenzlazo)quinoline (Solid phase)	0.18 µg/L	pH dependent worked between 5–7 pH only	[52]
1-Benzothiazole-2-yl-3(pyridine-3-ylazo)-nathalen-2-ol	8.5 µM	Lack of specificity, selective for AcO^−^, Hg^2+^, Cu^2+^, and electrochemically for Zn^2+^	[53]
Ninhydrin-thiosemicarbazone	1 µM	Dual sensing for Hg^2+^ and F^−^	[54]
2-(Anthracen-9-ylmethylene-hydrazonomethyl)-quinolin-8-ol	220 nM	pH dependent, worked between 6–8 pH only	[55]
Antipyrene Schiff base Silatranes	100 µM	Dual selectivity for Fe^3+^ and Hg^2+^	[56]
Silica nano-composite (11)	41.2 nM		Present work
Titania nano-composite (12)	18.8 nM		Present work

**Table 2:** BET analysis and CHN data of the SiO_2_ **(5)**, APTES@SiO_2_ **(9)**, **(3)**@APTES@SiO_2_ **(11)** and TiO_2_ **(6)**, APTES@TiO_2_ **(10)**, **(3)**@APTES@TiO_2_ **(12)**.

Entry	Elemental Analysis			BET Surface Area (m^2^/g)
	C%	H%	N%	
SiO_2_ **(5)**	-	-	-	201.81
APTES@SiO_2_ **(9)**	2.53	0.84	0.96	113.21
(3)@APTES@SiO_2_ **(11)**	4.52	0.98	1.32	77.56
TiO_2_ **(6)**	-	-	-	210.21
APTES@TiO_2_ **(10)**	5.65	1.29	1.87	95.35
**(3)**@APTES@TiO_2_**(12)**	19.24	1.05	1.42	60.43

**Table 3:** Quantitative analysis of an environmental sample with spiked amount of toxic Hg^2+^ ions using nano-composites **(11)** and **(12)**.

Nature of Sample Studied ^#^	Amount of Hg^2+^ Ions Added (nM)	Amount of Hg^2+^ Ions Found (nM) (*n* = 3 *)	Recovery %Age of Hg^2+^ Ions	Relative Error %Age
Nano-composite **(11)**				
Tap water	100	98.12	98.12	0.0010
	150	148.50	99.00	0.0028
	200	198.65	99.325	0.0024
Distilled water	100	99.30	99.30	0.0062
	150	149.48	99.65	0.0103
	200	199.24	99.62	0.0194
Bottled water	100	98.97	98.97	0.0085
	150	147.36	98.24	0.0056
	200	199.34	99.67	0.0064
Nano-composite **(12)**				
Tap water	100	99.67	99.67	0.0058
	150	149.28	99.52	0.0056
	200	199.21	99.61	0.0107
Distilled water	100	99.96	99.96	0.0124
	150	149.53	99.69	0.0091
	200	199.93	99.97	0.0105
Bottled water	100	99.67	99.67	0.0095
	150	149.42	99.61	0.0098
	200	199.89	99.94	0.0091

* *n* = Number of times test conducted, ^#^ = samples were taken from Punjab Agricultural University premises.

## Data Availability

Not applicable.

## Supplementary Materials

The following are available online at https://www.mdpi.com/article/10.3390/nano11113082/s1, Figure S1: (a) ^1^H NMR of ligand **(3)**. (b) D_2_O exchanges ^1^H NMR of ligand **(3)**. (c) ^13^C NMR of ligand **(3)**. (d) Mass spectra of ligand **(3)**; Table S1: EDX% of the elements present in the nano-composites **(11)**; Table S2: EDX% of the elements present in the nano-composites **(12)**.
