# Supplementary material for: Fluorescence Based Comparative Sensing Behavior of the Nano-Composites of SiO2 and TiO2 towards Toxic Hg2+ Ions
Source: Nanomaterials (Basel). 2021 Nov 15;11(11):3082. doi: 10.3390/nano11113082 (PMC8621696; doi:10.3390/nano11113082)
Supplement: Supplementary file 1 [file nanomaterials-11-03082-s001.zip › nanomaterials-1433837-SI.pdf]

Supplementary Materials

Fluorescence Based Comparative Sensing Behavior of the Nano-Composites of SiO_2 and TiO_2 towards Toxic Hg^{2+} Ions

Ekta and Divya Utreja*

Department of Chemistry, Punjab Agricultural University, Ludhiana 141004, Punjab, India; iektamalhotra@gmail.com

* Correspondence: utrejadivya@pau.edu Tel.: +91-9463852716

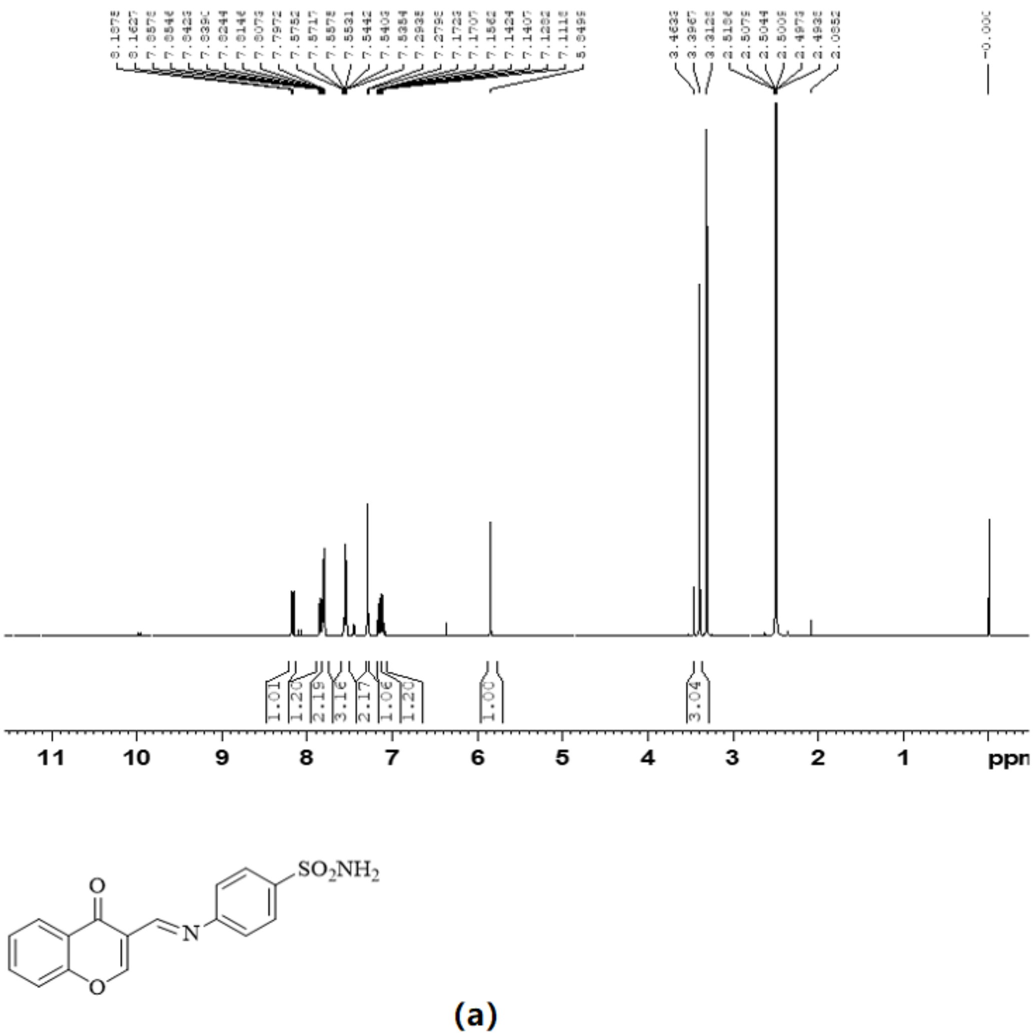

(a)

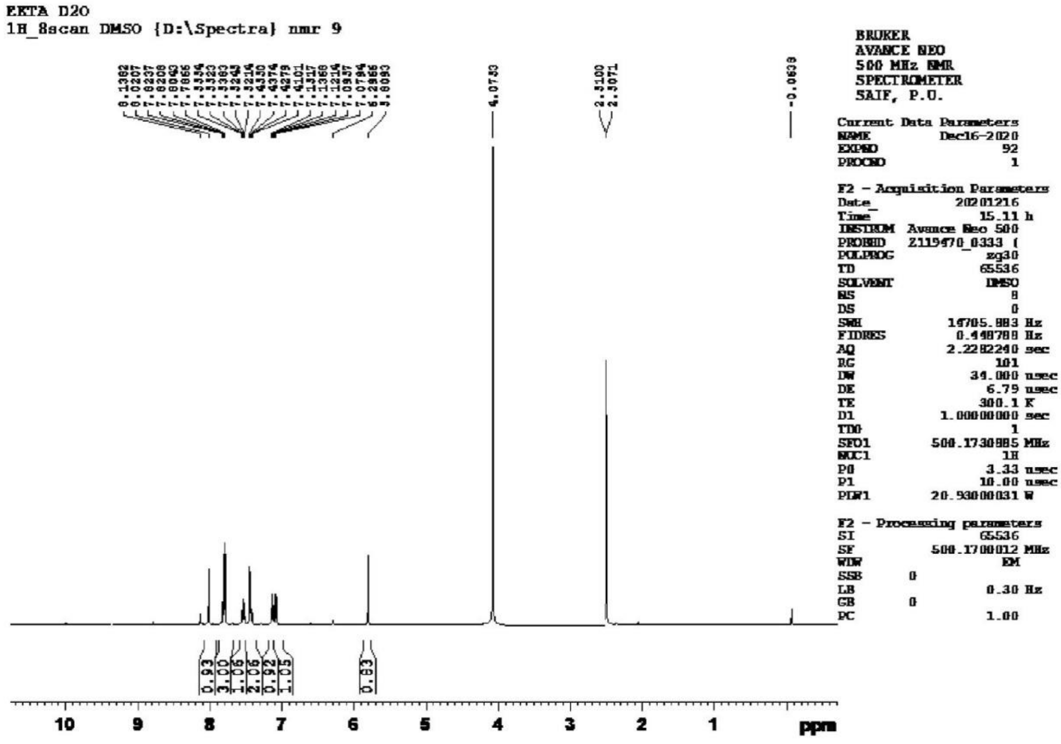
EKTA D2O
1H_8scan DMSO {D:\Spectra} nmr 9
BRUKER
AVANCE NEO
500 MHz NMR
SPECTROMETER
SAIF, P.U.
Current Data Parameters
NAME Dec16-2020
EXPNO 92
PROCNO 1
F2 - Acquisition Parameters
Date_ 20201216
Time 15.11 h
INSTRUM Avance Neo 500
PROBHD Z119470_0333 (
PULPROG zg30
TD 65536
SOLVENT DMSO
NS 8
DS 0
SWH 14705.883 Hz
FIDRES 0.448788 Hz
AQ 2.2282240 sec
RG 101
DW 34.000 usec
DE 6.79 usec
TE 300.1 K
D1 1.00000000 sec
TD0 1
SFO1 500.1730885 MHz
NUC1 1H
P0 3.33 usec
P1 10.00 usec
PLW1 20.93000031 W
F2 - Processing parameters
SI 65536
SF 500.1700012 MHz
WDW EM
SSB 0
LB 0.30 Hz
GB 0
PC 1.00
2.5100
2.5071
0.93
3.00
1.06
2.06
0.92
1.05
0.83
10
9
8
7
6
5
4
3
2
1
ppm

(b)

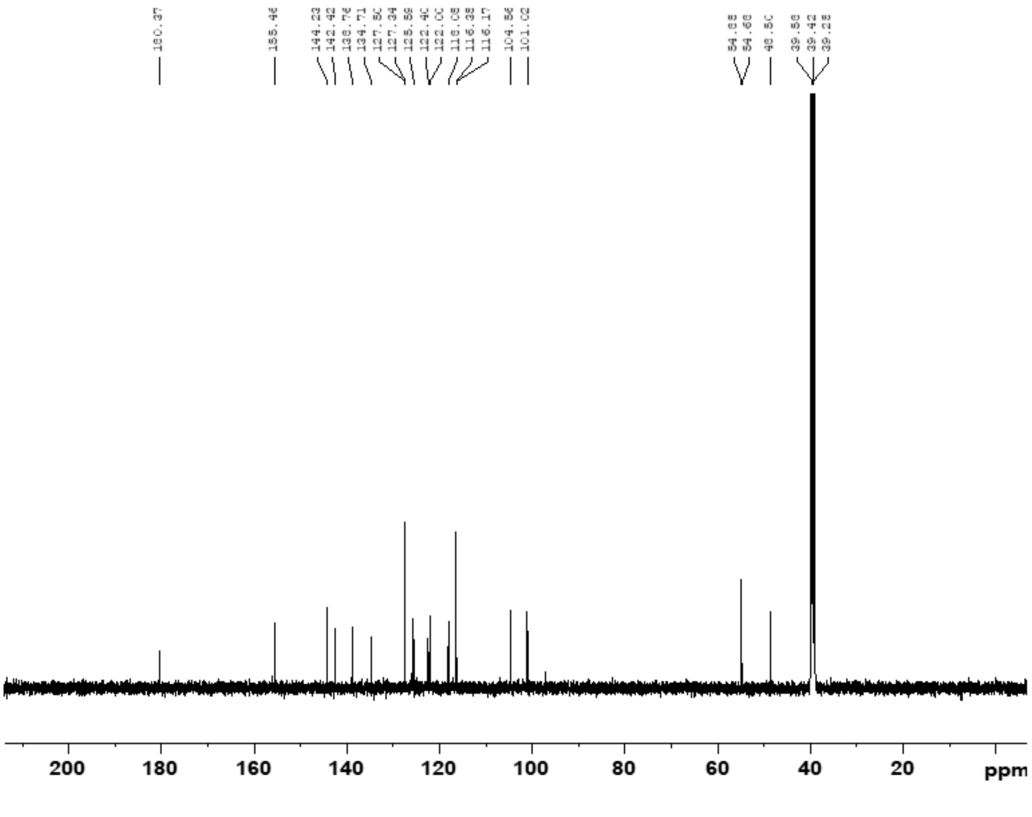
180.37
155.46
144.23
142.42
138.76
134.71
127.50
127.34
125.59
122.40
122.00
118.08
116.38
116.17
104.56
101.02
54.88
54.68
48.50
39.58
39.42
39.28
200
180
160
140
120
100
80
60
40
20
ppm

(c)

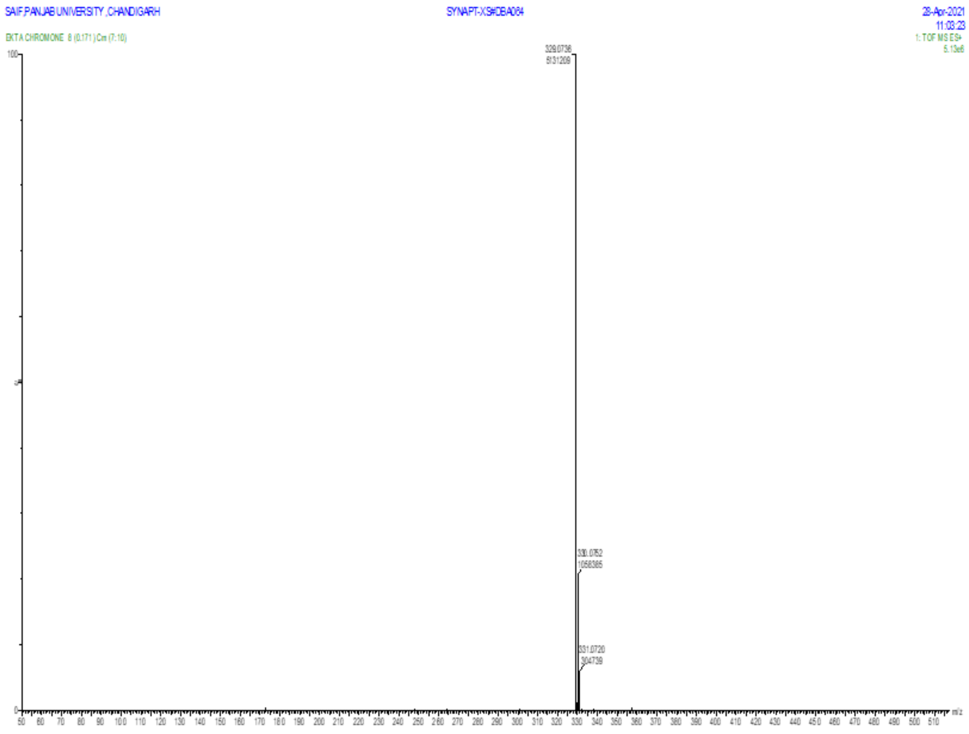

(d)

Figure S1. **(a)** ^{1}H NMR of ligand **(3)**. **(b)** D_2O exchanges ^{1}H NMR of ligand **(3)**. **(c)** ^{13}C NMR of ligand **(3)**. **(d)** Mass spectra of ligand **(3)**

Table S1: EDX % of the elements present in the organic-inorganic nano-composites **(11)**

**Element**	**Series [wt.%]**	**Un-normalized C [wt.%]**	**normalized C [wt.%]**	**Atom. [at.%]**	**C Error (3 Sigma)**
Oxygen	K-series	45.62	53.71	63.26	17.14
Silicon	K-series	33.41	39.33	26.39	4.27
carbon	K-series	4.44	5.22	8.19	3.50
nitrogen	K-series	1.28	1.51	2.03	1.39
Sulfur	K-series	0.20	0.23	0.14	0.12
Total		84.94	100.00	100.00	

Table S2: EDX % of the elements present in the organic-inorganic nano-composites **(12)**

**Element**	**Series [wt.%]**	**Un-normalized C [wt.%]**	**normalized C [wt.%]**	**Atom. [at.%]**	**C Error (3 Sigma)**
Carbon	K-series	19.51	19.90	31.46	13.10
Oxygen	K-series	45.55	46.46	55.13	28.09
Titanium	K-series	32.88	33.54	13.30	3.56
Sulfur	K-series	0.03	0.03	0.02	0.11
Nitrogen	K-series	0.07	0.07	0.10	0.67
Total		98.04	100.00	100.00	

1. Binding chemistry of probe (3) with Hg^{2+} ions

The stoichiometry ratio of the probe **(3)** (10 μM) towards Hg^{2+} (10 μM) was accompanied from emission based Job's plot. A sequence of solutions of complex **(3)+Hg^{2+}** were prepared with various ratios of **(3)**:**[Hg^{2+}]** to obtain the Job's plot with constant concentration of 10 μM. A scatter of emission intensity against mole fraction of probe **(3)** and **Hg^{2+}** ions complex showed maximum emission intensity at 0.5 mole fractions, which indicated 1:1 stoichiometry of probe **(3)** with analytes **Hg^{2+}** ions as shown in Figure. S2.

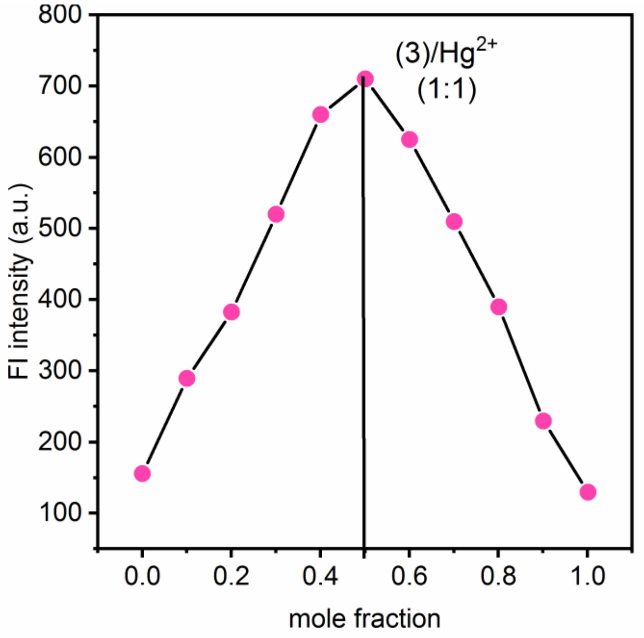

Figure S2: Job's plot for determination of the stoichiometry of probe **(3)+Hg^{2+}** in ACN/H_2O (1:1, *v*/*v*)

2. Density Functional Theory studies

DFT experiment was carried out by employing Gaussian 09 program software to optimize the 3D structure of the probe **(3)** and its complex **[(3)+Hg^{2+}]** B3LYP/6-311G and B3LYP/LANL2DZ were taken as basic sets, respectively. On studying the HOMO and LUMO orbitals of probe **(3)**, it was indicated that the more charge density was initially present over the chromone rings (fluorophore) of ligand in HOMO, which was later transferred to imines' and sulfonamide moiety of ligand in LUMO. Therefore, the fluorophore chromone is acting like donor site in molecule and sulfanilamide unit as acceptor site of ligand and electrons are transferring from oxygen atom of the chromone to the nitrogen atom of sulfanilamide moiety. Upon complexation with Hg^{2+} ion, more electron density was distributed in the HOMO of the fluorophore chromone and metal ion and which was further shifted over the chromone rings of complex in LUMO. Hence the HOMO-LUMO energy gap studies

confirmed the plausible binding of the Hg^{2+} ions with ligand **(3)**, which also interfere in the internal charge transfer (ICT) of ligand **(3)** and caused change in photo-physical properties and confirm the ligand to metal charge transfer (Figure. S3).

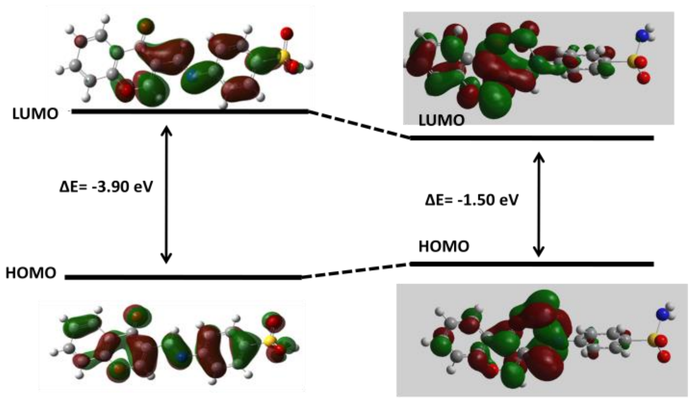

Figure S3: Theoretical evaluation of ligand **(3)** and complex **[(3)+Hg^{2+}]** (left to right)
